# Blood flow restriction training promotes functional recovery of knee joint in patients after arthroscopic partial meniscectomy: A randomized clinical trial

**DOI:** 10.3389/fphys.2022.1015853

**Published:** 2022-10-13

**Authors:** Junjie Ke, Xuchang Zhou, Yajing Yang, Hai Shen, Xiaobing Luo, Hui Liu, Lu Gao, Xin He, Xin Zhang

**Affiliations:** ^1^ Sichuan Provincial Orthopedic Hospital, Chengdu, China; ^2^ School of Sports Medicine and Health, Chengdu Sport University, Chengdu, China; ^3^ School of Sport Medicine and Rehabilitation, Beijing Sport University, Beijing, China; ^4^ National Cancer Center/National Clinical Research Center for Cancer/Cancer Hospital and Shenzhen Hospital, Chinese Academy of Medical Sciences and Peking Union Medical College, Shenzhen, China

**Keywords:** blood flow restriction training, meniscal injury, arthroscopic partial meniscectomy, isokinetic muscle strength test, physiotherapy, knee, exercise

## Abstract

**Purpose:** To explore the effect of blood flow restriction training (BFRT) on the recovery of knee function in patients after arthroscopic partial meniscectomy (APM).

**Methods:** Forty patients undergoing APM surgery were included in this parallel group, two-arm, single-assessor blinded, randomized clinical trial. The subjects were randomly divided into two groups: routine rehabilitation group (RR Group, *n* = 20) and routine rehabilitation + blood flow restriction training group (RR + BFRT Group, *n* = 20). One subject in each group dropped out during the experiment. All patients received 8 weeks of routine rehabilitation starting from the second day after APM. In addition, patients in the RR + BFRT group required additional BFRT twice a week. Visual analogue scale (VAS) score, range of motion (ROM), one-leg standing test (OLST) score, Lysholm knee score, quadriceps muscle strength, quadriceps thickness, and thigh circumference were evaluated at preoperative, postoperative, 4 and 8 weeks after surgery. SPSS 25.0 software was used for statistical analysis of the data. Repeated measures ANOVA was used if the data were normally distributed and had homogeneity of variance. Generalized estimating equations were chosen if the data were not normally distributed or had homogeneity of variance.

**Results:** There were no significant differences in VAS score, ROM, OLST score, Lysholm knee score, quadriceps muscle strength, quadriceps thickness, and thigh circumference between the two groups before surgery (*p* > 0.05). Compared with postoperative, VAS score, ROM, OLST score, Lysholm knee score, and thigh circumference were significantly improved in the RR group (*p* < 0.05), while quadriceps muscle strength and quadriceps thickness were not significantly enhanced at 8 weeks postoperatively (*p* > 0.05). However, VAS score, ROM, OLST score, Lysholm knee score, quadriceps muscle strength, quadriceps thickness, and thigh circumference were all significantly improved in the RR + BFRT group at 8 weeks postoperatively (*p* < 0.05). Furthermore, compared with the RR group, VAS score (50% vs. 86%), ROM (7.9% vs. 16.0%), OLST score (57.3% vs. 130.1%), Lysholm knee score (38.4% vs. 55.7%), relative peak torque (11.0% vs. 84.7%), mean power (20.6% vs. 88.1%), rectus femoris thickness (0.40% vs. 13.0%), vastus medialis (0.29% vs. 5.32%), vastus lateralis (0% vs. 6.2%), vastus internus (0% vs. 5.8%), and thigh circumference (2.7% vs. 5.8%) in the RR + BFRT group were significantly improved at 4 and 8 weeks postoperatively (*p* < 0.05).

**Conclusion:** BFRT combined with routine rehabilitation training can better promote the recovery of knee joint function in patients after APM, especially the improvement of quadriceps muscle strength and thickness.

## Introduction

Meniscus tear is one of the most common sports injuries to the knee joint, with an average annual incidence of 9.0 for men and 4.2 for women per 10,000 population (Cavanaugh, 2014). The meniscus is a very important fibrocartilage tissue in the knee joint, which mainly plays the role of buffering stress, absorbing oscillation, and maintaining the stability of joint movement. However, the knee meniscus is prone to injury in response to strong external shocks, such as fast turns and sharp stops in basketball (Flandry and Hommel, 2011). An arthroscopic partial meniscectomy (APM) is one of the commonly used clinical strategies for meniscal tears, which can improve the knee function of patients to a certain extent (Li et al., 2020). However, patients after APM are often accompanied by complications such as pain, atrophy of the knee extensor muscle group, and limited joint mobility. Importantly, it has been reported that the weakening of knee muscle strength in patients may persist for more than 4 years after surgery, which seriously affects the recovery of knee function and the quality of daily life (Ericsson et al., 2006). Therefore, patients after APM are usually required to undergo routine rehabilitation training. However, there are still some deficiencies in the routine rehabilitation training of patients after APM. For example, to effectively prevent muscle atrophy after APM, resistance exercise of appropriate intensity is required. The resistance training load that reaches more than 70% of the one-repetition maximum (1RM) can effectively increase muscle strength, which is difficult for patients after APM (American College Of Sports, 2009; Schoenfeld, 2013a). Patients after APM have lower knee muscle strength and poor functional activity, which may cause secondary injuries such as swelling, pain, and muscle fiber tearing during rehabilitation training. In addition, patient compliance may be hindered by pain caused by high-intensity training, resulting in the unsatisfactory effect of routine rehabilitation training. Blood flow re-striction training (BFRT) may be a potential strategy to compensate for this deficiency in routine rehabilitation training. BFRT was pioneered by SATO in Japan (Abe et al., 2006), which enables compression of the limb through compression cuffs to reduce arterial inflow and block venous outflow during rehabilitation training (Tegtbur et al., 2020). Jack et al. (2022) compared the efficacy of BFRT with conventional rehabilitation in patients after anterior cruciate ligament reconstruction (ACLR). The results showed that patients after ACLR in BFRT group had comparable outcome measures of muscle strength and bone loss to those in conventional rehabilitation group at 12 weeks postoperatively. Importantly, the time to RTS (return to sport) was significantly improved in BFRT group. In addition, Noyes et al. (2021) performed BFRT combined with low-resistance exercise at 30% of 1RM in patients with severe quadriceps and hamstring strength deficits after knee arthroscopy. They found significant improvements in measures of peak quadriceps and hamstring torque in the experimental group. Further, Hughes et al. (2019) performed BFRT combined with low-resistance training in ACLR patients undergoing unilateral hamstring autograft transplantation. It was found that the beneficial effects of BFRT combined with low-resistance training on muscles are similar to those of traditional high-intensity resistance training, resulting in skeletal muscle hypertrophy and improved knee extensor strength. Notably, patients in BFRT combined with low-resistance training group also experienced an overall improvement in physical function with greater reductions in knee pain and fluid accumulation, possibly due to the accumulation of metabolites in the limb under hypoxic conditions during BFRT performed at 80% limb occlusion pressure (LOP) (Scott et al., 2015; Lambert et al., 2018). Previous studies have shown that BFRT combined with low-resistance exercise increases systemic blood lactate, calcium, muscle-derived myokine, and blood acidity like high-intensity resistance exercise, resulting in enhanced muscle hypertrophy and bone anabolism (Fujita et al., 2007; Fry et al., 2010; Pearson and Hussain, 2015; Lambert et al., 2018). Thus, cumulative studies have demonstrated that BFRT combined with low-intensity resistance training, which can achieve muscle-beneficial effects similar to traditional high-intensity resistance training, has a favorable effect in promoting muscle strength and muscle hypertrophy in patients after ACLR (Hughes et al., 2017; Centner et al., 2019). However, the role of BFRT in the recovery of knee function in patients after APM remains unclear. Whether BFRT can effectively supplement the insufficiency of routine rehabilitation training to restore knee muscle strength and improve knee function in patients after APM remains to be further studied. Therefore, we propose a hypothesis that combining BFRT with routine rehabilitation training can promote the recovery of quadriceps femoris strength and knee function in patients after APM, which will provide scientific evidence and reference for the application of BFRT to routine rehabilitation training after APM.

## Materials and methods

### Participants

**FIGURE 1:**
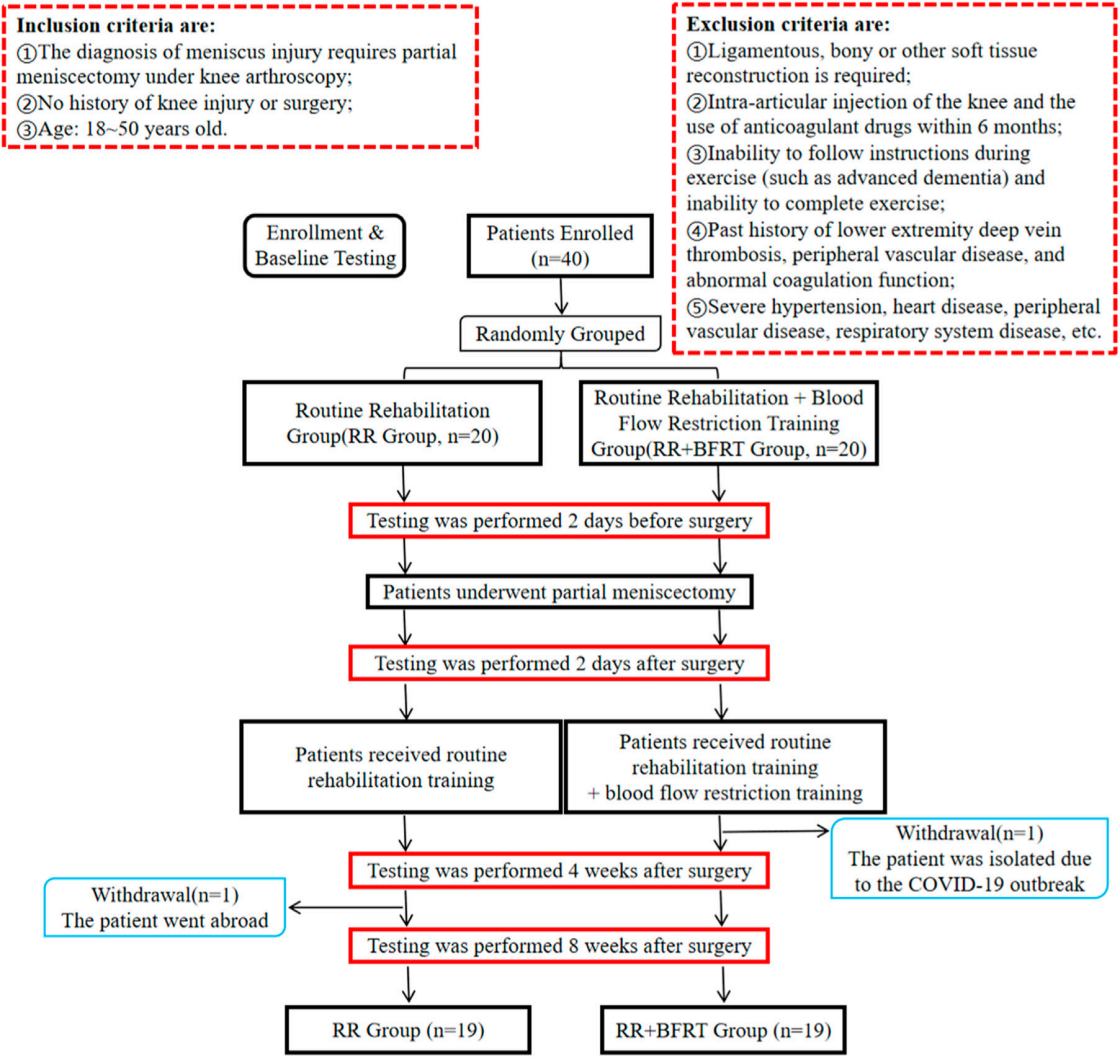
Flowchart overview of the study.

This study was approved and conducted in strict accordance with the recommendations from the Ethical Committee of Sichuan Provincial Orthopedic Hospital (KY2022-007-01). This study was a parallel group, two-arm, single-assessor blinded, randomized clinical trial in a between-subject’s repeated measures design. According to the inclusion and exclusion criteria, 40 patients who were to undergo APM were recruited from the Knee Trauma Department of Sichuan Orthopaedic Hospital between 1 July 2021 and 30 September 2021 (as shown in Figure 1). All patients were randomized into 2 groups: the routine rehabilitation group (RR Group, *n* = 20) and the routine rehabilitation + blood flow restriction training group (RR + BFRT Group, *n* = 20). Specifically, 10 opaque envelopes were prepared in advance. There are 4 folded slips inside each envelope, including 2 coded number 0 (coded 0 for RR Group) and 2 coded number 1 (coded 1 for RR + BFRT Group). Subjects were asked to randomly draw slips by an independent member of our research group who was not involved in subject recruitment and data collection analysis. The first four subjects were selected from envelope 1, and the sequence was followed until the last four subjects were selected from envelope 10. Considering the particularity of the rehabilitation training program, although the therapists and subjects were not blinded, the evaluators and data analysts were not aware of the specific groupings. All subjects voluntarily signed informed consent after being informed of the testing procedures and the possible risks during rehabilitation training. One subject in each group dropped out during the experiment. Ultimately, 19 patients in the RR Group and 19 patients in the RR + BFRT Group completed the trial. No adverse events occurred throughout the experiment. The characteristics of all patients are shown in Table 1. There were no statistically significant differences in baseline indicators such as gender, age, height, weight, BMI, surgical side, injury time, limb occlusion pressure (LOP), and 1RM between the two groups (*p* > 0.05).

**TABLE 1:** Patient characteristics.

Variable	RR group	RR + BFRT group
Sex (male/female)	9/10	12/7
Age (yrs)	37.74 ± 11.27	37.58 ± 11.44
Height (cm)	164.84 ± 9.33	167.05 ± 8.30
Body mass (kg)	63.24 ± 13.08	67.63 ± 9.68
BMI (kg/m^2^)	23.08 ± 3.23	24.18 ± 2.72
Injured leg (left/right)	6 ± 13	7 ± 12
Injury time (mon)	8.22 ± 8.60	7.14 ± 7.15
LOP (mmHg)	171.95 ± 16.29	173.68 ± 14.70
1RM (kg)	58.06 ± 21.89	60.94 ± 20.71

### Sample size calculating

G*Power 3.1 software was used to calculate the required sample size in our study (Hughes et al., 2019). The F model was used to achieve 95% power at α = 0.05. The specific calculation parameters are as follows: effect size = 0.32, power = 0.95, a total of 2 groups, and the number of repeated measurements is 4 times. The calculation result is *n* = 34. Furthermore, considering that there may be a 17% churn rate during the trial, we finally determined that the total sample size to be included in the experiment was 40. According to the ratio of 1:1, there are 20 subjects in each group.

### Routine rehabilitation

All patients in the RR group and RR + BFRT group received routine rehabilitation training from day 2 postoperatively, twice a week for 8 weeks. According to the literature (Abe et al., 2006; Tegtbur et al., 2020; Jack et al., 2022) and our previous clinical experience, the routine rehabilitation training program was formulated: 1) 5 min warm-up exercise: including self-pulling and free walking-running on the elliptical machine; 2) Active ROM training of the lower extremity: sliding bed exercises for active knee flexion and extension, 10 repetitions per set, 10 s rest between sets, a total of 3 sets; 3) Squat training within the 0–90° motion arc: 10 repetitions per set, 10 s each time, 30 s rest between sets, a total of 3 sets; 4) Walking training for 5 min; 5) The German GYM80 intelligent strength training system was used to perform low-intensity pedaling closed-chain training within the range of motion of the knee joint from 0 to 90°. During muscle contraction, concentric and eccentric contractions alternate in rhythmic cycles of 1 s each. The pedaling training load is 30% of 1RM. Each set was repeated 30, 15, 15 and 15 times in turn with a 30 s interval between sets, a total of 4 sets; 6) Ankle pump exercise and ice compress: 3 sets of 10 repetitions with 30 s rest between sets.

### Blood flow restriction training

**FIGURE 2:**
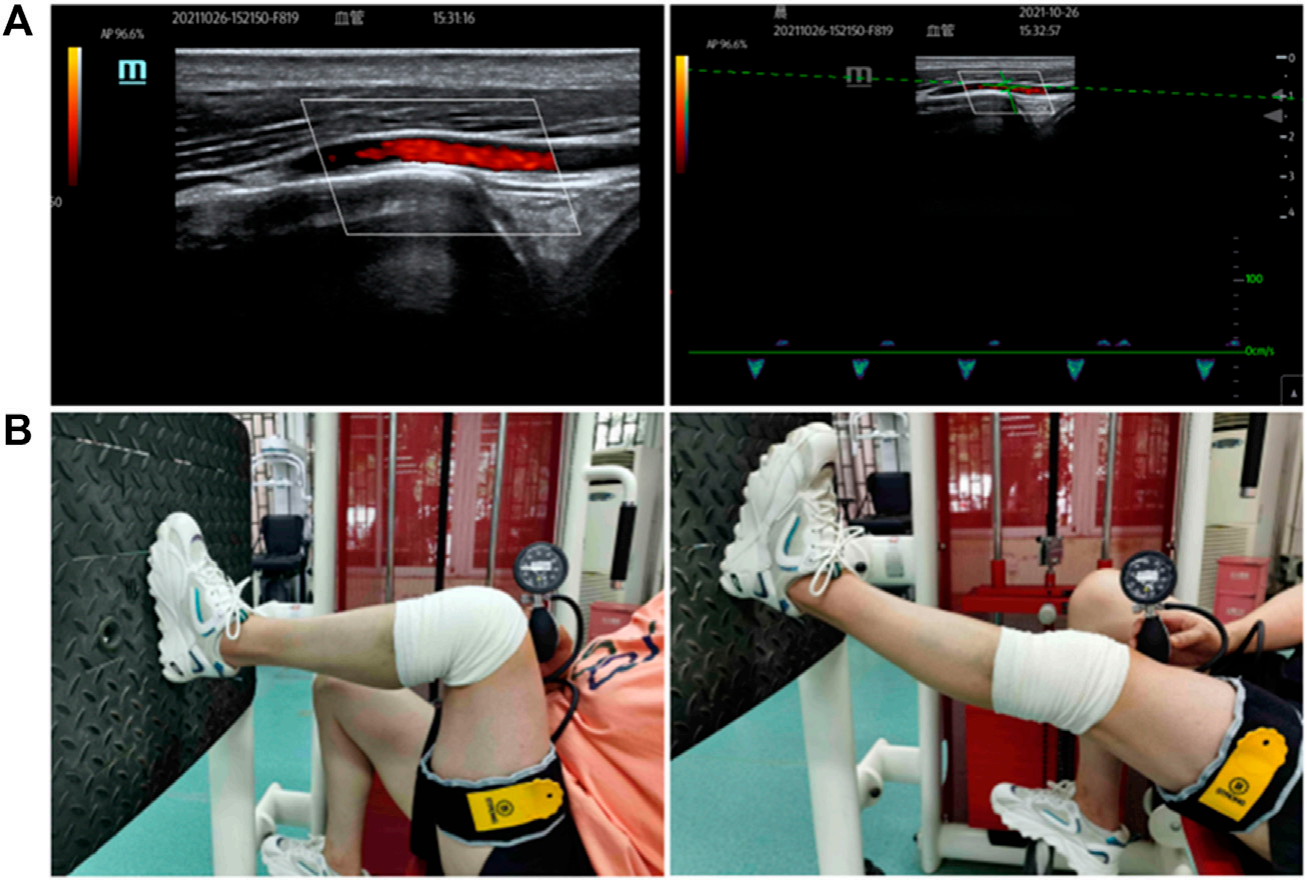
Schematic diagram of blood flow restriction training [**(A)**: ultrasound was used to measure limb occlusion pressure; **(B)** live image of blood flow restriction training].

Patients in the RR + BFRT group were required to undergo BFRT during routine resistance training. A portable automated tourniquet system (Delfi Medical, Vancouver, BC, Canada), equipped with a tourniquet of corresponding size, was used to partially occlude blood vessels during rehabilitation training. The same width of cuff, a dual-purpose easy-fit variable contour nylon cuff (11.5 cm × 86 cm, 5 mm thick), was used for all patients, as determined by the manufacturer’s recommendations. Despite changes in muscle volume during rehabilitation training, the system allows precise control of cuff pressure to ensure constant LOP. According to the previous literature (Lambert et al., 2018; Hughes et al., 2019; Noyes et al., 2021), the BFRT program was formulated: 1) Since the operative knee cannot support excessive loads, 1RM was accurately predicted by measuring 10RM under the protection of the physiotherapist; 2) An inflatable cuff is attached to the proximal thigh and slowly inflated. Ultrasound was used to detect the pulse of the dorsal artery of the foot (As shown in Figure 2). The cuff pressure was recorded when the dorsal artery pulse disappeared, which was defined as LOP. The 80% LOP value was chosen as the cuff pressure for BFRT; 3) The tourniquet system was applied to the base of the subject’s thigh to restrict blood flow while performing low-intensity pedaling closed-chain training, which is consistent with the training regimen of the RR group; 4) There is a 30 s rest period between sets. The tourniquet remains inflated and pressurized throughout the training session. The tourniquet can be inflated for a maximum of 5 min, including the rest time between sets.

### Quadriceps muscle strength

A multi-joint isokinetic strength testing and training system (CON-TREX, Switzerland, model: CON-TREX ^®^MJ + TP1000) was used to evaluate quadriceps muscle strength. Relative peak torque and mean power values were selected to assess quadriceps muscle strength (Scott et al., 2015). The relative peak torque reflects the highest point of the torque curve, which can evaluate the absolute strength of the lower limb weight-bearing muscle group. Mean power refers to the average amount of work per unit time, which can reflect the actual work efficiency of the muscles. Subjects in the seated position were fastened with wide straps on their upper body and thighs. Subjects were required to grip the handles on either side of the test chair, keeping the backrest at an 85° angle to the horizontal. A resistance pad connected to the power device was fixed 3 cm above the subject’s lateral malleolus, while the axis of the power device’s axis of rotation was aligned with the center of the knee joint (lateral femoral condyle). Lower extremities were weighed to eliminate the influence of lower extremity weights. The test was performed within the 0–90° arc of motion of the knee joint. The test speed is 60°/s, 5 times per set. 30 s of continuous passive motion (CPM) is required as a warm-up to prevent knee injury.

The visual analogue scale/score (VAS) was used to assess the degree of knee pain in patients. The total score is 10 points. The higher the score, the more severe the pain. 0 points represent no pain, and 10 points represent extreme pain.

### Quadriceps thickness

**FIGURE 3:**
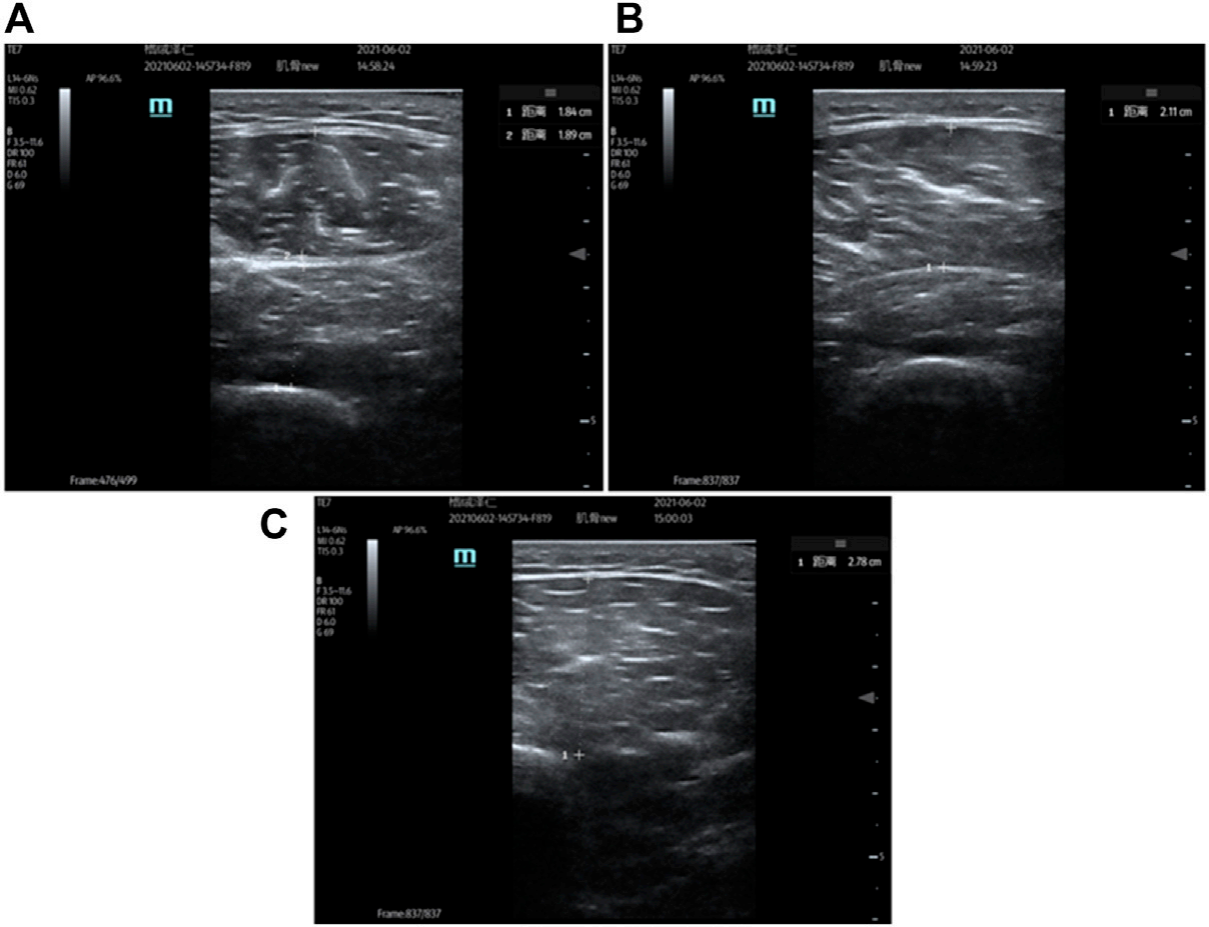
Ultrasound measurement of quadriceps thickness [**(A)**: ultrasound measurement of the rectus femoris and vastus intermedius; **(B)** ultrasound measurement of the vastus lateralis; **(C)** ultrasound measurement of the vastus medialis].

A SuperSonic MACH 40 ultrasound imaging system (Hologic, America) from the Department of Radiology was used to measure the thickness of the quadriceps (Fry et al., 2010). Subjects were required to lie supine on a portable treatment couch. A 4.2–13.0 MHz broadband linear array scanning transducer (12.7 mm × 47.1 mm) lubricated with coupling agent was gently placed on the marked area of the thigh skin without pressing on the skin surface. Tissue deformation that can be caused by excessive compression is eliminated by observing that no tissue motion occurs in the live ultrasound image. When a clear image with visible aponeurosis and individual nerve bundles is displayed on the screen, “freeze” the image and save it for further analysis. Muscle thickness (cm) was defined as the distance between the superficial and deep aponeurosis at the widest point in each image (as shown in Figure 3). The average was calculated from three images. The specific marking positions are as follows: 1) The thickness of the rectus femoris and vastus intermedius was measured on the acquired ultrasound image at 50% of the distance from the anterior superior iliac spine to the superior border of the patella; 2) A mark was made at 50% of the distance from the anterior superior iliac spine to the superior border of the patella to measure the circumference of the thigh. Subsequently, the thickness of the vastus lateralis was measured at the point where the marked point was shifted laterally by 1/10 of the circumference on the acquired ultrasound images; 3) A mark was made at 20% of the distance from the anterior superior iliac spine to the superior border of the patella to measure the circumference of the thigh. Subsequently, the thickness of the vastus medialis was measured at the point where the marked point was shifted laterally by 1/8 of the circumference on the acquired ultrasound images.

### Thigh circumference

Thigh circumferences are measured by a trained physiotherapist using a standard tape measure (Chen et al., 2020). The subjects were instructed to keep their muscles relaxed by placing their feet fully on a flat surface while gently flexing their knees. Subsequently, circumference is measured at the midpoint level of the lateral surface of the thigh, midway between the femoral tuberosity and the lateral top of the tibia. All measurements were performed by the same physiotherapist. The average of two measurements was taken as the result.

### Pain

The visual analogue scale/score (VAS) was used to assess the degree of knee pain in patients. The total score is 10 points. The higher the score, the more severe the pain. 0 points represent no pain, 10 points represent extreme pain.

### Knee function

Lysholm knee scoring scale was used to assess the patient’s knee function (Hughes et al., 2019). The Lysholm Knee Scoring System consists of 8 questions: limp, pain, support, locking, swelling, instability, stair-climbing, and squatting. The score is 0 (worst result) to 100 (best result). The lower the score, the smaller the functional limitations. A total score of 95 or more is considered excellent; 94–85 is considered good; 84–65 is considered pass; less than 65 is considered poor.

### Balance function

One-leg standing test (OLST) was used to assess patients’ balance function (Woon et al., 2021). Subjects with hands on hips were required to stand on one leg while the other leg was raised with the hip flexed and the knee bent at 90°. Postural control ability and time spent maintaining posture while standing were recorded.

### ROM

A dedicated protractor was used to measure range of motion (ROM). Subjects, lying supine on the treatment couch, were required to actively bend the knee and slide the heel as far as possible toward the buttocks. Subsequently, a protractor was used to measure the maximum flexion and extension of the patient’s knee. Three measurements were averaged to assess the ROM of the knee. All stages of testing were performed by the same experienced assessor to minimize measurement errors.

### Statistical analysis

SPSS 25.0 software was used for statistical analysis of the data. If the measurement data conformed to a normal distribution, the mean ± standard deviation (mean ± SD) was used for description; if not, the median (interquartile range) was used for description. If the enumeration data met the conditions of the X^2^ test, the X^2^ test was used; otherwise, the Fisher’s exact probability method was used. If the measurement data satisfied normality and homogeneity of variance, one-way ANOVA was used; if not, the Kruskal-Wallis H test was used. The comparison of repeated measures data was as follows: 1) Repeated measures ANOVA was used if the data were normally distributed and had homogeneity of variance. If the data satisfied the sphericity test, the within-subject effect test was used; if not, the multivariate test was used. If the interaction effect was significant, the analysis of the simple effect was selected; if not, the corresponding main effects analysis was selected. 2) Generalized estimating equations were chosen if the data were not normally distributed or had homogeneity of variance. If the interaction effect was significant, a simple effects analysis was performed. *p* < 0.05 represents a significant difference.

## Results

### BFRT enhances quadriceps muscle strength in patients after APM

**FIGURE 4:**
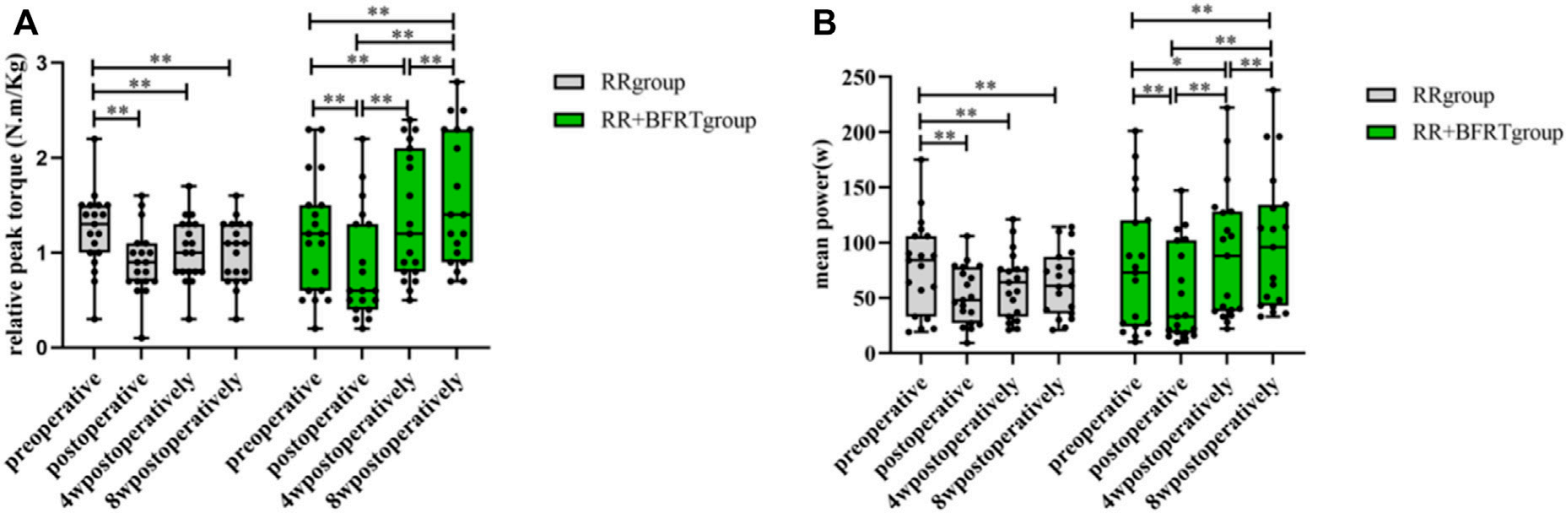
Intra-group comparison of relative peak torque and mean power in RR and RR + BFRT groups. “**” indicates a significant change (*p* < 0.01). Values are presented as mean ± SD. RR Group: routine rehabilitation group; RR + BFRT Group: routine rehabilitation + blood flow restriction training group. [**(A)**: Changes in relative peak torque in the RR group and the RR + BFRT group; **(B)** Changes in mean power in the RR group and the RR + BFRT group].

**FIGURE 5:**
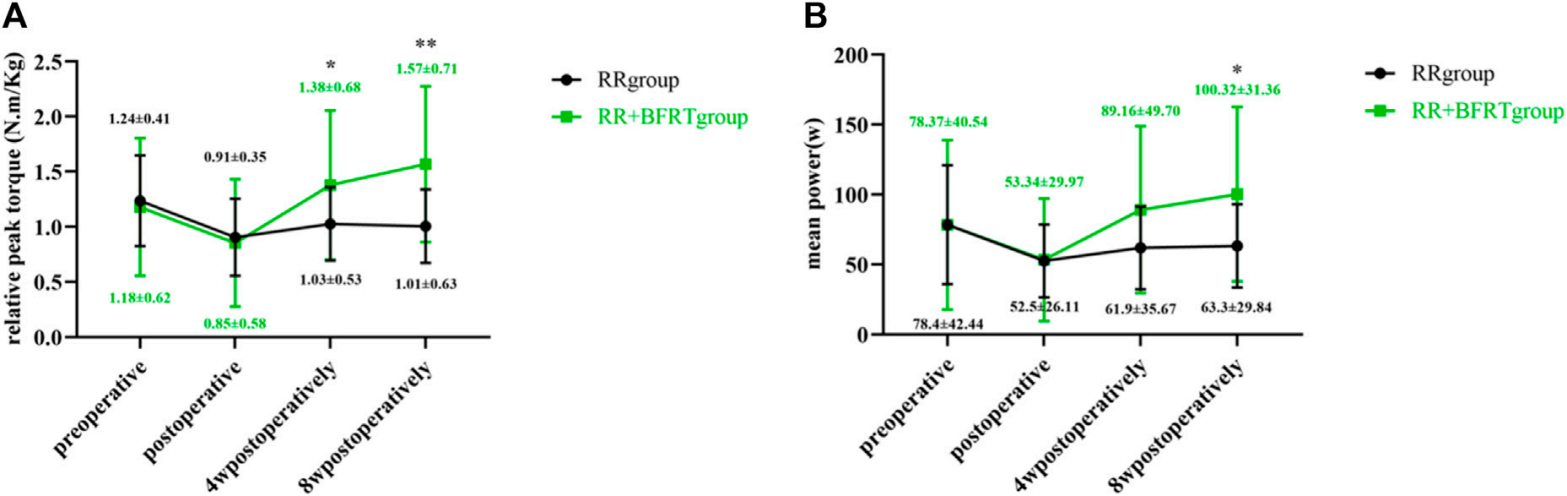
Comparison of relative peak torque and mean power between RR group and RR + BFRT group at different time points. “*” indicates a significant change (*p* < 0.05). “**” indicates a significant change (*p* < 0.01). Values are presented as mean ± SD. RR Group: routine rehabilitation group; RR + BFRT Group: routine rehabilitation + blood flow restriction training group. [**(A)**: Comparison of relative peak torque between RR group and RR + BFRT group; **(B)** Comparison of mean power between RR group and RR + BFRT group].

Relative peak torque and mean power values were measured by the Isokinetic Strength Testing and Training System to assess quadriceps muscle strength in patients after APM. As shown in Figure 4, the relative peak torque and mean power of patients in the RR group and RR + BFRT group decreased significantly after surgery (*p* < 0.01). Furthermore, at 4 and 8 weeks after surgery, the relative peak torque and mean power of patients in the RR group did not significantly improve (*p* > 0.05), while the relative peak torque and mean power of patients in the RR + BFRT group were significantly improved (*p* < 0.01). In addition, there was no significant difference in the relative peak torque and mean power values between the RR group and the RR + BFRT group after surgery (*p* > 0.05). However, at 4 and 8 weeks after surgery, the relative peak torque and mean power values of patients in the RR + BFRT group were significantly higher than those in the RR group (*p* < 0.05) (as shown in Figure 5), which indicates that BFRT could significantly enhance the quadriceps muscle strength in patients after APM.

### BFRT increases quadriceps thickness in patients after APM

**FIGURE 6:**
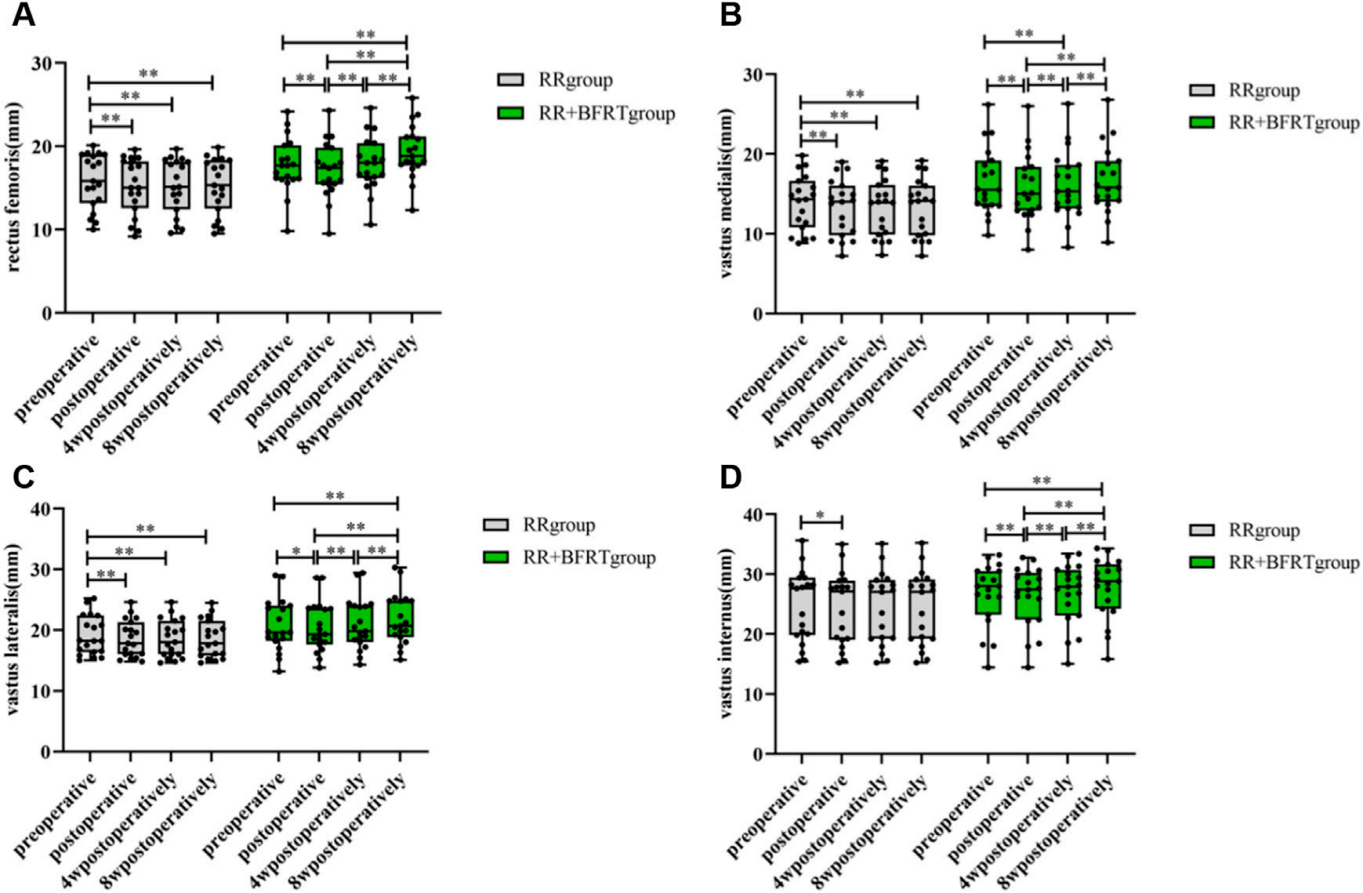
Intra-group comparison of quadriceps thickness in RR and RR + BFRT groups. “*” indicates a significant change (*p* < 0.05). “**” indicates a significant change (*p* < 0.01). Values are presented as mean ± SD. RR Group: routine rehabilitation group; RR + BFRT Group: routine rehabilitation + blood flow restriction training group. [**(A)**: Changes in rectus femoris thickness in the RR group and the RR + BFRT group; **(B)** Changes in vastus medialis thickness in the RR group and the RR + BFRT group; **(C)** Changes in vastus lateralis thickness in the RR group and the RR + BFRT group; **(D)** Changes in vastus intermedius thickness in the RR group and the RR + BFRT group].

**FIGURE 7:**
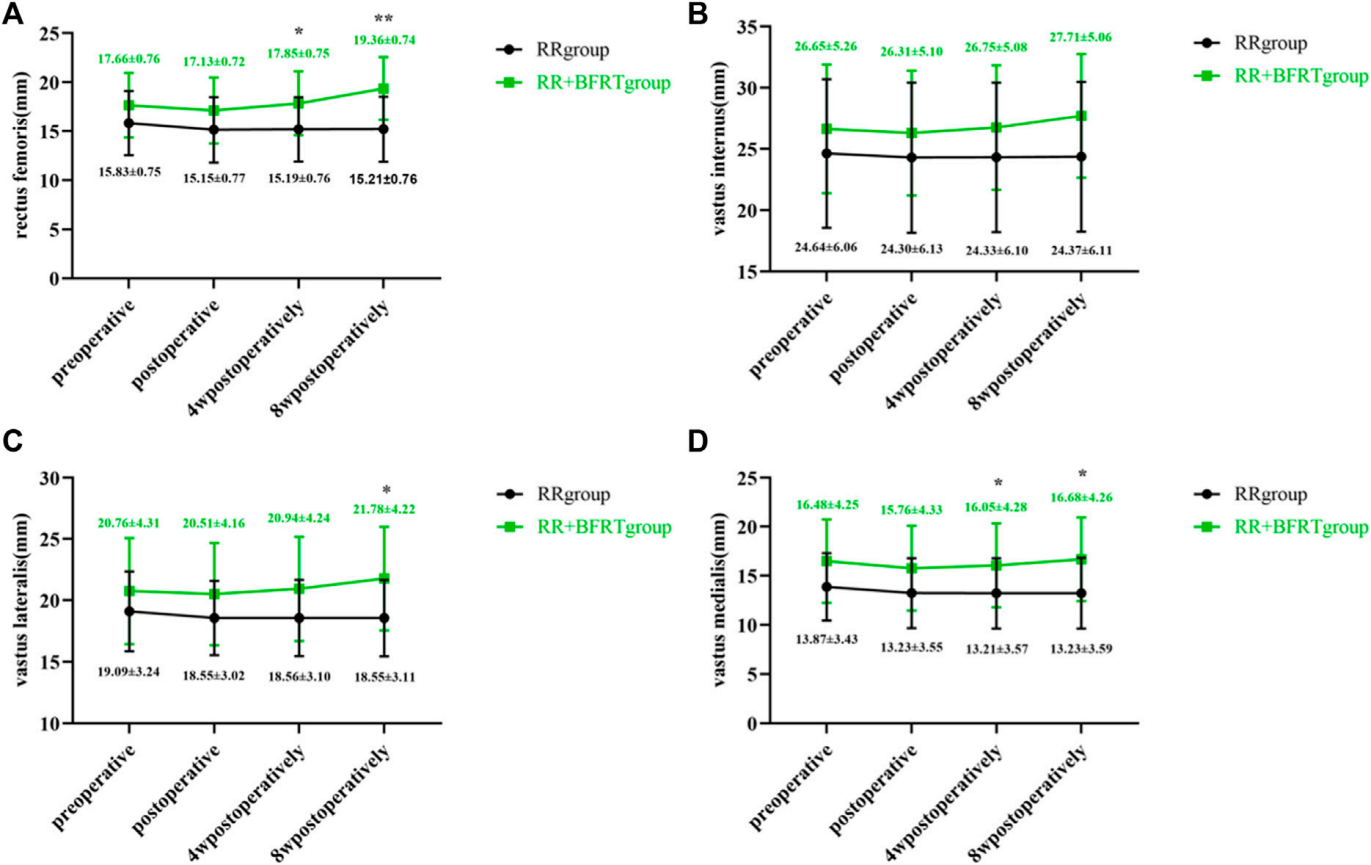
Comparison of quadriceps thickness between RR group and RR + BFRT group at different time points (mm). “*” indicates a significant change (*p* < 0.05). “**” indicates a significant change (*p* < 0.01). Values are presented as mean ± SD. RR Group: routine rehabilitation group; RR + BFRT Group: routine rehabilitation + blood flow restriction training group. [**(A)**: Comparison of rectus femoris between RR group and RR + BFRT group; **(B)** Comparison of vastus internus between RR group and RR + BFRT group; **(C)** Comparison of vastus lateralis between RR group and RR + BFRT group; **(D)** Comparison of vastus medialis between RR group and RR + BFRT group].

Doppler color ultrasound was used to measure the thickness of the rectus femoris, vastus intermedius, vastus lateralis, and vastus medialis in patients after APM. As shown in Figure 6, the thicknesses of the rectus femoris (*p* < 0.01), vastus intermedius (*p* < 0.01), vastus lateralis (*p* < 0.01) and vastus medialis (*p* < 0.05) were significantly reduced in the RR group and the RR + BFRT group after surgery. At 4 weeks postoperatively, the thicknesses of the above muscles were significantly increased in the RR + BFRT group (*p* < 0.05). Importantly, the thickness of the quadriceps muscle continued to increase in the RR + BFRT group at 8 weeks postoperatively. However, the thickness of the quadriceps muscle of the patients in the RR group did not increase significantly (*p* > 0.05) either at 4 or 8 weeks after surgery. Further analysis found that there was no significant difference in the thickness of the rectus femoris, vastus intermedius, vastus lateralis and vastus medialis between the RR group and the RR + BFRT group after surgery (*p* > 0.05) (as shown in Figure 7). However, the thickness of rectus femoris, vastus intermedius and vastus lateralis (except vastus medialis) in RR + BFRT group were significantly better than those in RR group at 4 and 8 weeks after operation (*p* < 0.05). The above results suggest that BFRT can significantly increase quadriceps muscle thickness in patients after APM.

### BFRT increases thigh circumference in patients after APM

**FIGURE 8:**
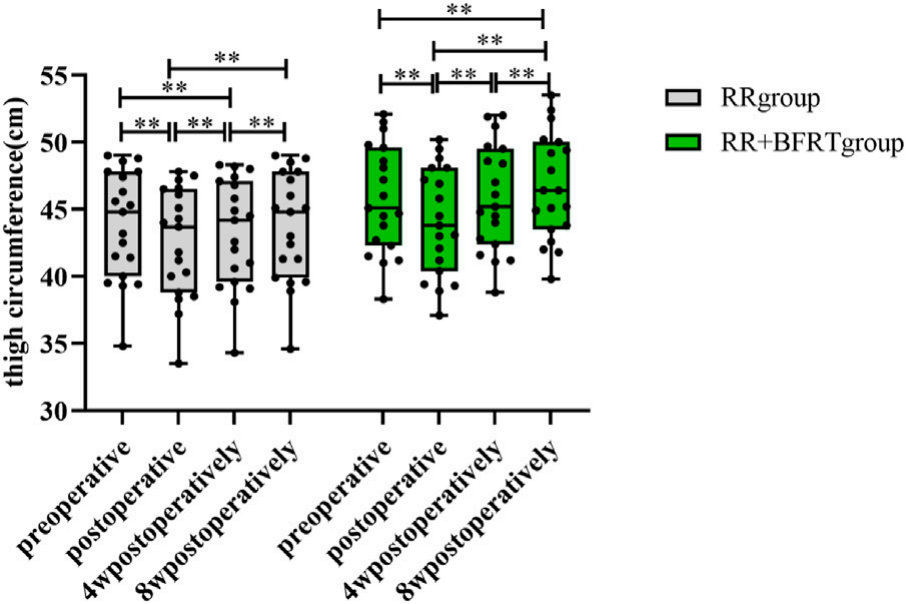
Intra-group comparison of thigh circumference in RR and RR + BFRT groups. “**” indicates a significant change (*p* < 0.01). Values are presented as mean ± SD. RR Group: routine rehabilitation group; RR + BFRT Group: routine rehabilitation + blood flow restriction training group.

**FIGURE 9:**
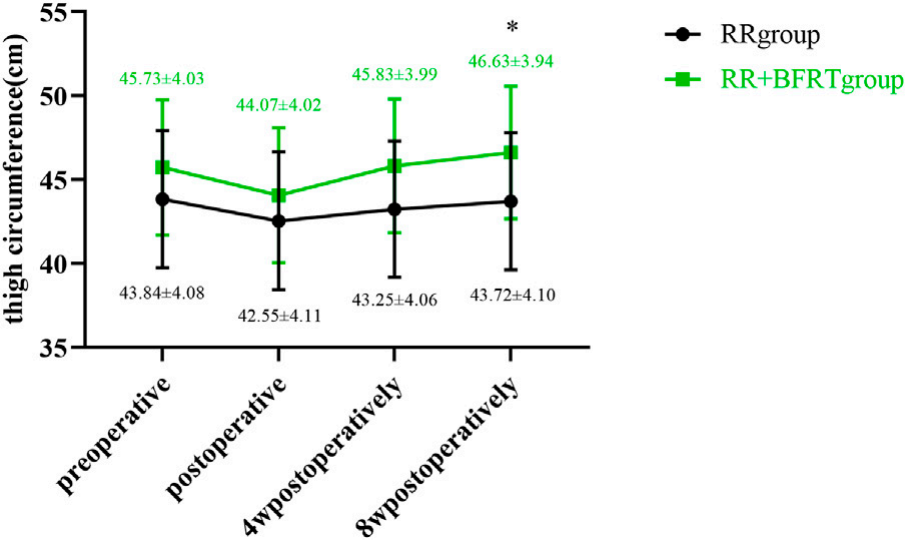
Comparison of thigh circumference between RR group and RR + BFRT group at different time points (cm). “*” indicates a significant change (*p* < 0.05). Values are presented as mean ± SD. RR Group: routine rehabilitation group; RR + BFRT Group: routine rehabilitation + blood flow restriction training group.

A standard tape measure was used to measure thigh circumference in patients after APM. As shown in Figure 8, both the RR group and the RR + BFRT group had a significant decrease in thigh circumference after surgery (*p* < 0.01). At 4 weeks postoperatively, the thigh circumference of patients in the RR group increased significantly (*p* < 0.01), although it was still lower than the preoperative thigh circumference. However, at 4 weeks postoperatively, the thigh circumference of the RR + BFRT group was significantly increased (*p* < 0.01) and was significantly higher than the preoperative thigh circumference (*p* < 0.01). In addition, there was no significant difference in thigh circumference between the RR group and the RR + BFRT group after surgery (*p* > 0.05) (as shown in Figure 9). However, at 8 weeks postoperatively, the thigh circumference of the RR + BFRT group was significantly higher than that of the RR group (*p* < 0.05). It was shown that BFRT can significantly increase the thigh circumference of patients after APM.

### BFRT relieves knee pain in patients after APM

**FIGURE 10:**
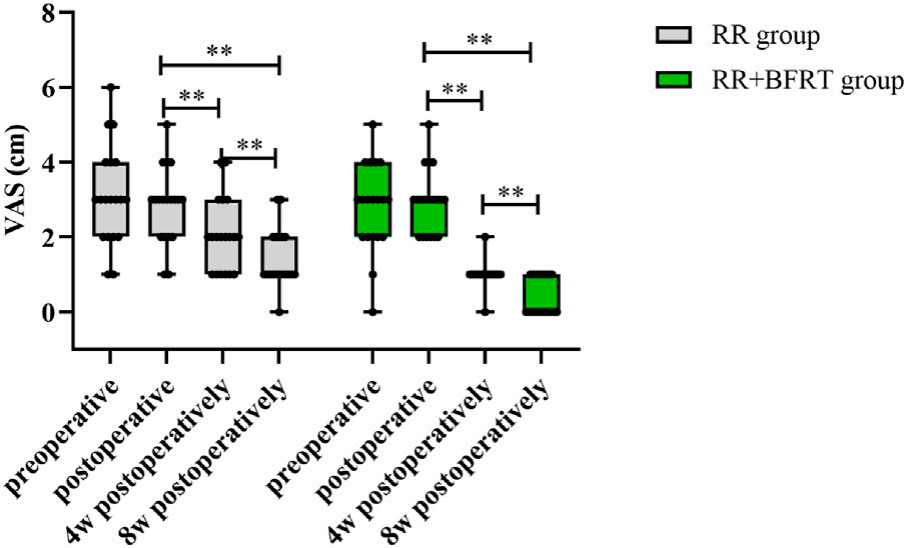
Intra-group comparison of VAS scores in RR group and RR + BFRT group. “**” indicates a significant change (*p* < 0.01). Values are presented as mean ± SD. RR Group: routine rehabilitation group; RR + BFRT Group: routine rehabilitation + blood flow restriction training group.

**FIGURE 11:**
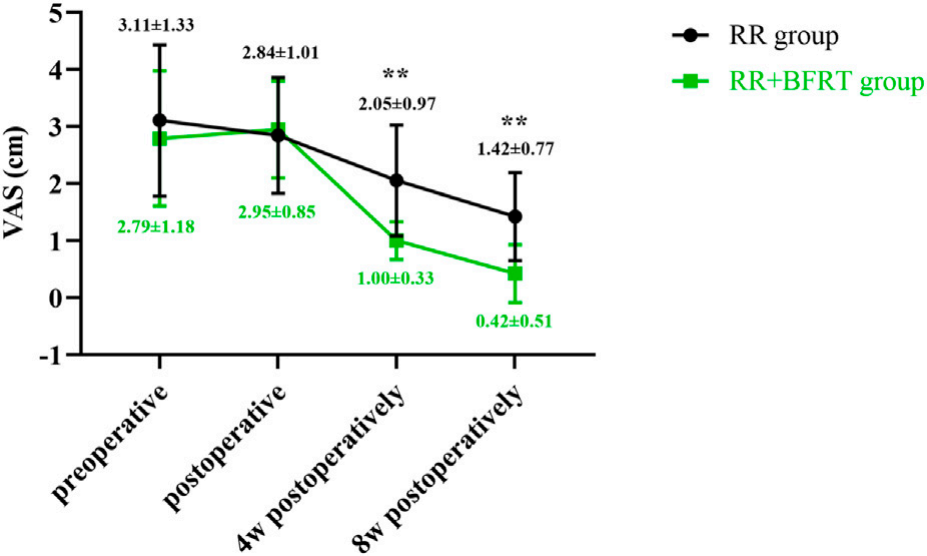
Comparison of VAS scores between RR group and RR + BFRT group at different time points (cm). “**” indicates a significant change (*p* < 0.01). Values are presented as mean ± SD. RR Group: routine rehabilitation group; RR + BFRT Group: routine rehabilitation + blood flow restriction training group.

The VAS score was used to evaluate the degree of knee pain in patients after APM. The VAS scores of the RR group and the RR + BFRT group were significantly decreased at 4 and 8 weeks postoperatively (*p* < 0.01) (as shown in Figure 10). As shown in Figure 11, there was no statistical difference in the VAS scores of patients in the RR group and the RR + BFRT group at preoperative and postoperative (*p* > 0.05). However, further analysis found that the VAS score of the RR + BFRT group was significantly lower than that of the RR group at 4 and 8 weeks postoperatively (*p* < 0.01). The above results suggest that BFRT can significantly relieve knee pain in patients after APM.

### BFRT improves knee function in patients after APM

**FIGURE 12:**
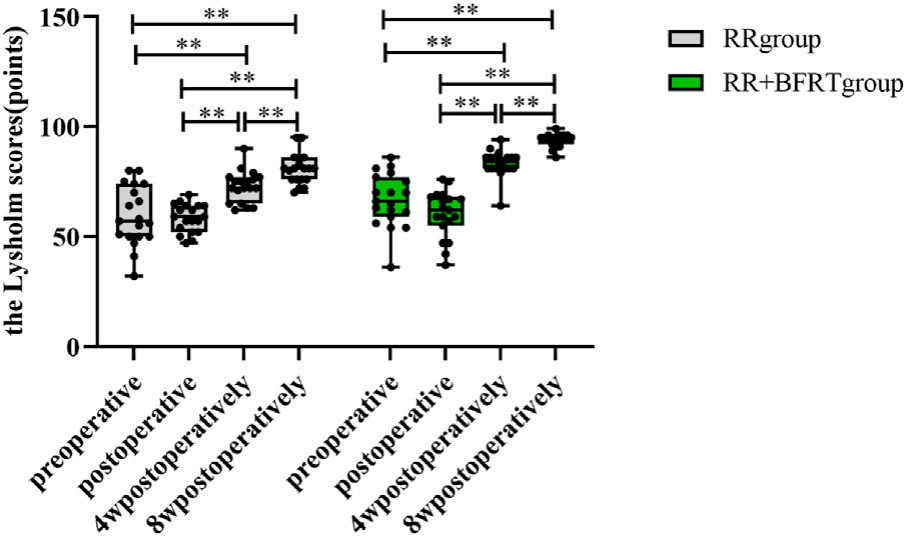
Intra-group comparison of Lysholm scores in RR group and RR + BFRT group. “**” indicates a significant change (*p* < 0.01). Values are presented as mean ± SD. RR Group: routine rehabilitation group; RR + BFRT Group: routine rehabilitation + blood flow restriction training group.

**FIGURE 13:**
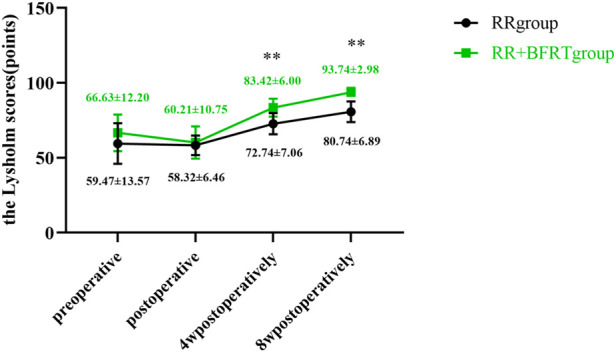
Comparison of Lysholm scores between RR group and RR + BFRT group at different time points (points). “**” indicates a significant change (*p* < 0.01). Values are presented as mean ± SD. RR Group: routine rehabilitation group; RR + BFRT Group: routine rehabilitation + blood flow restriction training group.

The Lysholm score was used to assess knee function in patients after APM. As shown in Figure 12, compared with preoperative, there was no significant difference in the Lysholm score between the RR group and RR + BFRT group after the operation (*p* > 0.05). Lysholm scores of the patients in the RR group and the RR + BFRT group were significantly improved at 4 and 8 weeks after surgery (*p* < 0.01). Further analysis found that Lysholm scores of patients in the RR + BFRT group were significantly higher than those in the RR group at 4 and 8 weeks after surgery (*p* < 0.01) (as shown in Figure 13), indicating that BFRT could significantly improve knee function of patients after APM.

### BFRT improves balance function in patients after APM

**FIGURE 14:**
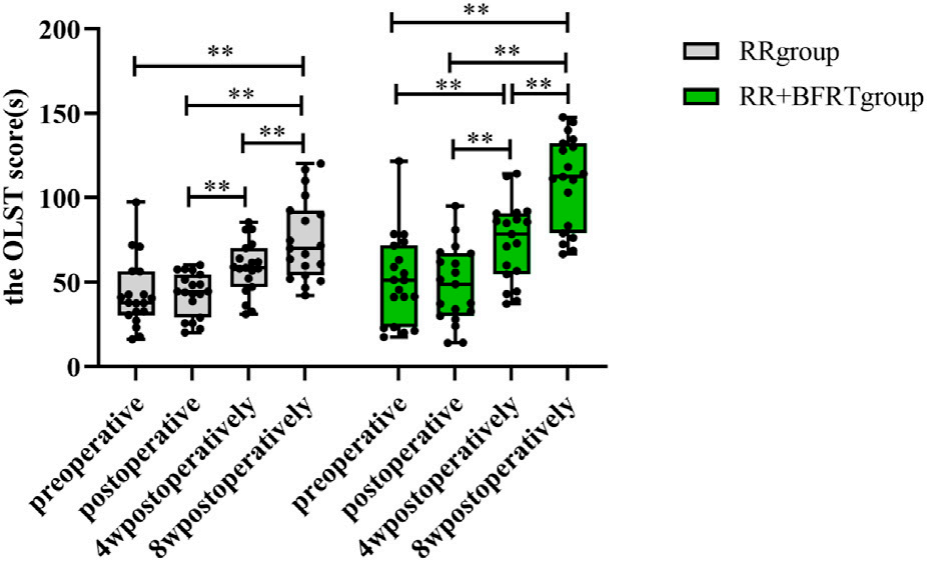
Intra-group comparison of OLST scores in the RR group and the RR + BFRT group. “**” indicates a significant change (*p* < 0.01). Values are presented as interquartile range. RR Group: routine rehabilitation group; RR + BFRT Group: routine rehabilitation + blood flow restriction training group.

**FIGURE 15:**
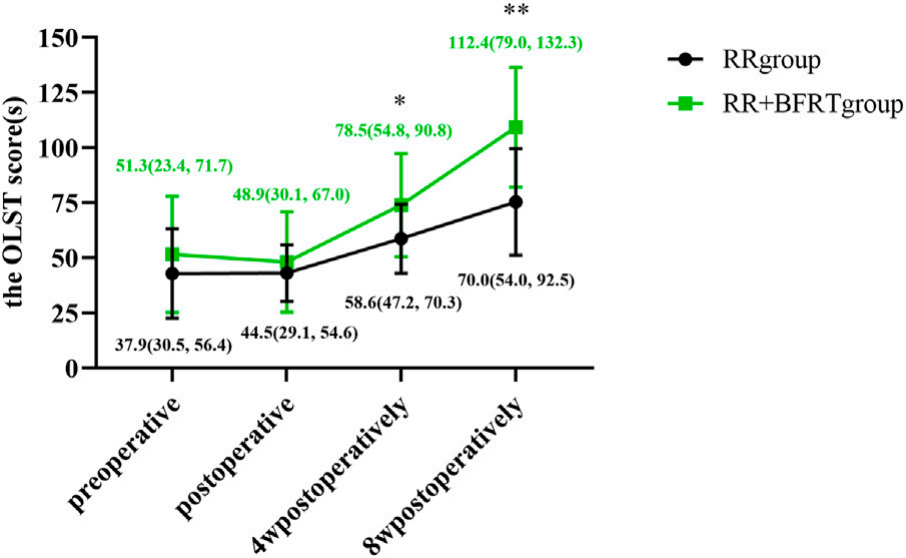
Comparison of OLST scores between RR group and RR + BFRT group at different time points (s). “*” indicates a significant change (*p* < 0.05); “**” indicates a significant change (*p* < 0.01). Values are presented as interquartile range. RR Group: routine rehabilitation group; RR + BFRT Group: routine rehabilitation + blood flow restriction training group.

The OLST score was used to assess balance function in patients after APM. As shown in Figure 14, compared with preoperative, there was no significant difference in the OLST scores of the RR group and the RR + BFRT group after surgery (*p* > 0.05). Compared with postoperative, the OLST scores of patients in the RR group and RR + BFRT group were significantly improved at 4 and 8 weeks postoperatively. However, although the OLST scores of both the RR group and the RR + BFRT group were significantly improved at 4 weeks postoperatively, the OLST score of the RR + BFRT group was significantly higher than that of the RR group (*p* < 0.05) (as shown in Figure 15). In addition, the OLST score of the RR + BFRT group was also significantly higher than that of the RR group at 8 weeks after surgery (*p* < 0.01). It indicates that BFRT can significantly improve the balance function of patients after APM.

### BFRT increases the range of motion of the knee joint in patients after APM

**FIGURE 16:**
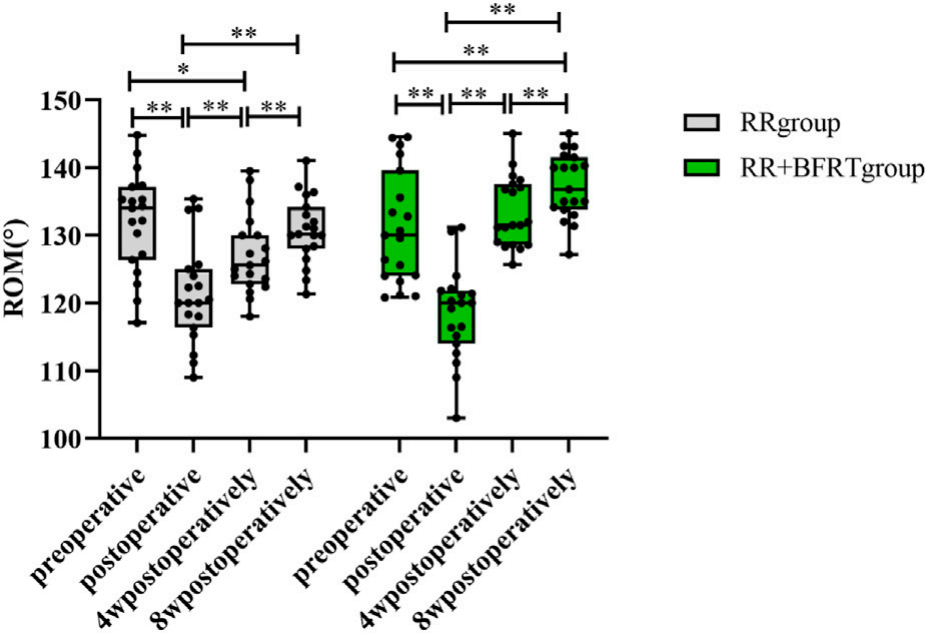
Intra-group comparison of knee ROM in RR group and RR + BFRT group. “**” indicates a significant change (*p* < 0.01). Values are presented as mean ± SD. RR Group: routine rehabilitation group; RR + BFRT Group: routine rehabilitation + blood flow restriction training group.

**FIGURE 17:**
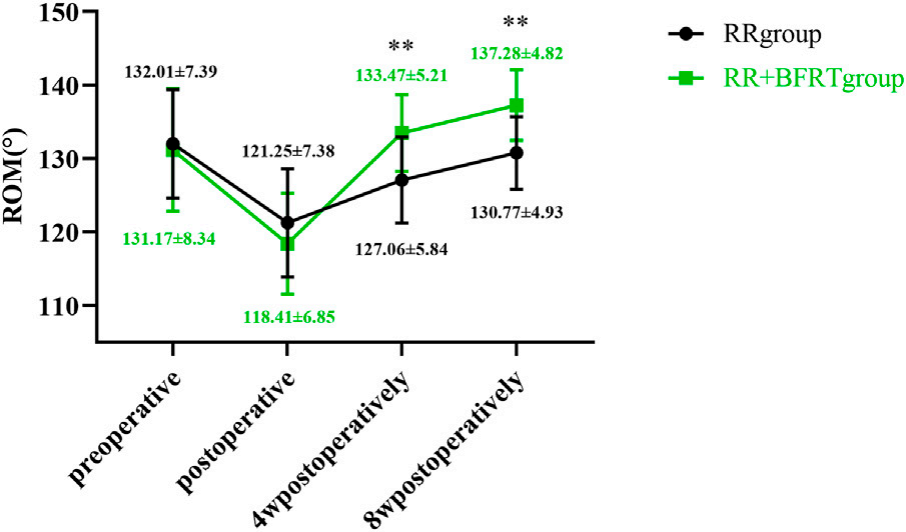
Comparison of knee ROM between RR group and RR + BFRT group at different time points (°). “**” indicates a significant change (*p* < 0.01). Values are presented as mean ± SD. RR Group: routine rehabilitation group; RR + BFRT Group: routine rehabilitation + blood flow restriction training group.

A joint-specific protractor was used to measure the ROM of the knee joint in patients after APM. As shown in Figure 16, the ROM of both the RR group and the RR + BFRT group was significantly decreased compared to preoperatively (*p* < 0.01). In the RR + BFRT group, compared with preoperative, there was no significant difference in knee ROM of patients at 4 weeks postoperatively (*p* > 0.05). However, in the RR group, there was a significant difference in knee ROM at 4 weeks postoperatively compared with preoperative (*p* < 0.01), although there was no significant difference at 8 weeks postoperatively (*p* > 0.05). In addition, the further comparison between groups found that the ROM in the RR + BFRT group was significantly higher than that in the RR group at 4 and 8 weeks postoperatively (*p* < 0.01) (as shown in Figure 17). It was shown that BFRT can significantly increase the ROM of the knee joint in patients after APM.

## Discussion

Knee meniscus tears are more common in athletes, manual workers, and the elderly with knee joint degeneration. APM is a widely accepted clinical strategy for meniscal tears to enhance knee stability and partially restore knee function, thereby alleviating the potential for severe secondary injury (Weinreb et al., 2017; Musahl and Karlsson, 2019). However, postoperative complications such as knee pain, limited mobility, muscle atrophy, and muscle weakness may occur (Cruz-Martinez et al., 2000; Akima and Furukawa, 2005), which is consistent with the findings of this study. Therefore, effective early postoperative rehabilitation training is essential for patients to recover knee function and return to a high-quality daily life. Patients after APM cannot fully tolerate resistance training loads of 70% and above of 1RM due to postoperative pain and joint dysfunction (American College Of Sports, 2009), which makes resistance training loads sufficient to reverse muscle weakness not available to the patient. In this study, BFRT was introduced as an emerging rehabilitation training strategy after knee APM, which can prevent muscle atrophy by promoting muscle strength and muscle hypertrophy with low-intensity load, enabling patients to return to pre-injury exercise levels as soon as possible. Our study found that compared with routine rehabilitation training alone, BFRT combined with routine rehabilitation training showed more significant positive effects in improving pain, ROM, balance function, knee function, quadriceps muscle strength, and thigh circumference in patients after APM.

Cumulative studies have shown that BFRT combined with low-intensity resistance training is as effective in enhancing muscle strength as high-intensity resistance training alone. Tennent et al. (2017) performed 2-week BFRT in patients after knee arthroscopy with the resistance of 30% 1RM and 80% LOP. The results showed that the thigh circumference and knee extensor strength were significantly improved in the BFRT group, which was consistent with our findings. In addition, Hughes et al. (2019) performed 8-week rehabilitation training in 28 patients with anterior cruciate ligament reconstruction. The study found that BFRT combined with low-intensity resistance training (30% of 1RM) was similar to high-intensity resistance training (70% of 1RM) in improving muscle strength and size. However, Curran et al. (2020) explored the effect of BFRT combined with high-intensity resistance training (70% 1RM) on the functional recovery of quadriceps in 34 patients with ACL reconstruction. The results showed that the quadriceps strength and muscle atrophy of the patients did not significantly improve after 8 weeks of rehabilitation training. It indicates that the combination of BFRT and high-intensity resistance training may not be necessary to improve quadriceps muscle function in patients after ACL reconstruction. In this study, we found significant improvements in quadriceps strength and thickness in patients after APM after 8 weeks of rehabilitation training. Interestingly, ROM and balance function were also significantly better in the RR + BFRT group compared with the RR group, indicating that combining BFRT with low-intensity exercise at 30% 1RM can also show beneficial training effects for patients after APM.

Knee extensor strength is one of the important factors for the normal function of the knee joint and the maintenance of body balance. A recent study showed that severe thigh muscle weakness in patients after knee arthroscopy was significantly improved after nine sessions of BFRT (Noyes et al., 2021). Our study also found that at 8 weeks after surgery, the relative peak torque and mean power of the quadriceps femoris in the RR + BFRT group were significantly increased compared with those before the operation and were significantly higher than those in the RR group, indicating that BFRT combined with routine rehabilitation training can significantly enhance the quadriceps strength in patients after APM, compared with routine rehabilitation training alone (Hughes et al., 2017; Lixandrao et al., 2018; Centner et al., 2019). Takano et al. (2005) found that short-term low-intensity training with the reduction of muscle blood flow can effectively promote the up-regulation of hormones such as growth hormone (HGH) related to muscle synthesis, which peaks at half an hour after exercise training. Other studies have also shown that BFRT causes an increase in HGH and insulin-like growth factor-1 (IGF-1) concentrations in the blood, which can promote the proliferation and differentiation of muscle satellite cells, and ultimately enhance muscle strength (Duan et al., 2010; Schoenfeld, 2013b). In addition, this study found that after 8 weeks of rehabilitation training, in addition to the enhancement of quadriceps strength, the balance function of patients after APM was also significantly improved. A possible explanation is that BFRT enhances neuromuscular control of the lower extremities by increasing proprioceptive input. However, it must be admitted that a few studies have reached conflicting conclusions: there was no significant difference in quadriceps strength changes in the BFRT group compared with the routine rehabilitation training group (Mason et al., 2022). The possible reason is that the exercise load of the participants was lower than 20%–30% of 1RM, resulting in an insufficient number of motor units devoted to muscle contraction, resulting in no significant increase in muscle strength. In addition, the physical therapist’s proficiency in the BFRT technique and the patient’s strict adherence to the training program are also factors that influence the success of rehabilitation training. Overall, our study shows that BFRT combined with low-intensity resistance training significantly improves quadriceps strength in patients after APM, which is consistent with most research findings (Slysz et al., 2016).

Muscle atrophy is common after knee injuries and surgery (Ross and Worrell, 1998). The thickening of muscle fibers plays an important role in enhancing its contractility and reactivity, increasing the basal metabolic rate, and delaying the decline of muscle function (Hoh, 1992). Takarada et al. (2000) found that patients after cruciate ligament reconstruction in the BFRT group had a nearly 50% reduction in extensor muscle atrophy compared to the control group. In our study, quadriceps thickness and thigh circumference were significantly increased in both RR and RR + BFRT groups at 8 weeks postoperatively. Moreover, patients in the RR + BFRT group showed a greater increase compared to the RR group. However, another study explored the effect of BFRT with a pressure value of 130–180 mmHg combined with quadriceps isometric and straight leg raise training in athletes after ACL reconstruction. It was found that there was no significant difference between the control and BFRT groups in quadriceps atrophy, suggesting that BFRT was not effective in preventing quadriceps muscle atrophy after ACL reconstruction in the athlete population (Iversen et al., 2016). The reason for the difference in the above results may be related to the training intensity lower than the minimum intensity (10% 1RM) necessary to prevent muscle atrophy. Moreover, the possible reason may also be that the training effect of isotonic training on the muscles of athletes is worse than that of ordinary people since athletes’ muscles are stronger than ordinary people. Previous studies have found that patients after knee arthroscopy cannot tolerate high-intensity resistance exercise or anaerobic exercise, resulting in reduced stimulation of type II muscle fibers and overall atrophy of the thigh muscles (Baugher et al., 1984). Some scholars have shown that the reason why BFRT combined with low-intensity resistance exercise can achieve the effect of traditional high-intensity exercise and improve postoperative muscle atrophy is that on the basis of low-intensity resistance training, restricted blood flow stimulates the preferential synthesis and recruitment of muscle hypertrophy and strength-related type II muscle fibers, resulting in muscle fiber hypertrophy and proliferation (Manini et al., 2011). Under normal circumstances, high-intensity resistance training greater than 70% of 1RM is required to achieve this exercise effect (Segal et al., 2015). Gundermann et al. (2014) found that the activity of the mTORC1 signaling pathway was enhanced and muscle protein synthesis was increased in muscle tissue of the BFRT group. At 24 h after BFRT, muscle protein synthesis was increased by 69.4% compared with that before training. However, since the 24-h post-exercise protein synthesis response occurs after feeding, it is difficult to determine if its BFRT or feeding paired with exercise. Further studies are needed to elucidate the underlying mechanisms that may be involved in muscle metabolic responses after acute BFRT. Furthermore, another study showed that BFRT can induce changes in muscle metabolism, which may be related to the acute release of anabolic hormones such as growth hormone (GH) (Burgomaster et al., 2003). On the one hand, the expression of insulin-like growth factor-1 (IGF-1) is promoted to increase muscle volume; on the other hand, the expression of vascular endothelial growth factor (VEGF) is promoted to stimulate angiogenesis to accommodate the greater demand for blood supply as muscle mass increases (Burgomaster et al., 2003). In our study, quadriceps thickness and overall thigh circumference were significantly increased in the RR + BFRT group compared with the RR group. The underlying mechanism of BFRT-induced quadriceps hypertrophy remains to be further elucidated. This study provides important insights for the optimization of rehabilitation training programs after APM and provides a reference for the mechanism research of BFRT to promote muscle hypertrophy.

The adverse effects of postoperative pain on the body are more serious than we know it. Postoperative pain not only causes negative emotions in patients such as insomnia and anxiety but also hinders early activities and functional exercise (Kehlet, 2018). Therefore, the effective relief of postoperative pain can not only make the patient more comfortable but more importantly, enable the early functional exercise to be carried out smoothly, thereby shortening the hospitalization time of the patient. Some studies have found that the continuous contraction of the cuff for 5–30 min may induce diffuse noxious inhibitory controls (DNIC) effects, which may be caused by noxious stimuli such as ischemic pain and resistance exercise-induced muscle pain (Fujii et al., 2006; Tuveson et al., 2006). These nociceptive stimuli cause nociceptive and convergent neurons in the same segment to transmit nociceptive information to the higher center. Subsequently, the nociceptive information transmitted to the higher center activates the DNIC through the endogenous analgesic system and descends to inhibit all convergent neurons that were not initially activated by noxious stimuli to prevent the input of various nociceptive and non-nociceptive information, ultimately acting as analgesic with pain (Le Bars et al., 1979). This study found that compared with the postoperative baseline value, the VAS value of patients in the RR + BFRT group decreased by 85.76% at 8 weeks after surgery, while the VAS value of patients in the RR group decreased by only 54.34% at 8 weeks after surgery, which indicates that BFRT combined with routine rehabilitation training has a greater benefit in relieving pain in patients after APM, compared with routine rehabilitation training alone. This finding is consistent with that of Hughes et al. (2018). Their study found that knee pain in patients with ACL reconstruction in the BFRT group was significantly lower than in the high-intensity training group. The possible reason is that BFRT combined with low-intensity resistance training is less difficult, resulting in higher patient compliance during rehabilitation training since the pain experienced by patients during BFRT is tolerable. Importantly, BFRT also helps relieve knee pain. Therefore, BFRT is suitable for pain relief in patients after knee arthroscopy.

Although the currently limited data show that BFRT is safe for postoperative rehabilitation training, no adverse events such as muscle injury, ecchymosis, swelling, and local tenderness were found in this experiment, suggesting that it is safe to apply BFRT for rehabilitation training after APM (Patterson et al., 2019). Ohta et al. (2003) reported 2 patients who dropped out during BFRT due to lower extremity discomfort or dull pain. Other studies reported no adverse events or did not report the presence or absence of adverse events during BFRT (Tennent et al., 2017). Some experts suggest that all patients should be screened for contraindications to BFRT before clinical application (Dephillipo et al., 2018). Overall, contraindication screening, individualized BFRT exercise prescription for patients, and appropriate medical supervision are needed before BFRT is administered.

## Limitations and perspectives

A BFRT combined with low-intensity resistance training can effectively increase quadriceps muscle strength and prevent muscle atrophy in patients after knee arthroscopy by restricting blood flow through a compression cuff. BFRT regimen may be an important factor in achieving changes in muscle hypertrophy. Although significant differences in short-term rehabilitation training on muscle strength and hypertrophy were found in our study, no long-term follow-up data were provided, making the long-term effects of BFRT on muscle mass unclear. Notably, there is currently no widely accepted protocol for measuring muscle mass in BFRT-related studies. Further research is needed to optimize measures of muscle mass. In addition, it must be acknowledged that some of the intervenient variables may influence the outcomes in this study. On the one hand, because the subjects were not hospitalized for rehabilitation training, the physical activity of subjects at home was not effectively monitored and managed, which may have affected the outcome variables. On the other hand, due to some uncontrollable factors, such as COVID-19 epidemic and schedule conflicts, two subjects ended their rehabilitation training ahead of schedule. Furthermore, since this study is a single-assessor blinded, randomized clinical trial, the single-blind method cannot avoid the subjective bias brought by the investigators. Moreover, in this study, the formulation of the BFRT scheme was mainly based on the previous literature, and the optimal BFRT scheme was not explored. The optimal parameters for BFRT to achieve muscle hypertrophy remain unclear. Further research is needed to determine the optimal BFRT training regimen, including pressure values, duration, training load, number of repetitions, cuff width, etc. The ideal BFRT protocol ensures that restrictive pressures are set for each limb to maximize clinical outcomes and patient compliance. In addition, another limitation of this study is that 1RM in patients after APM was not remeasured as muscle strength and size increased during the 8-week rehabilitation training. In our experiments, patients were required to persist in going to the outpatient clinic for rehabilitation training. Patients were reluctant to undergo re-measurement of 1RM because they felt that it would have no significant benefit to their recovery but added an additional training burden. It is recommended that further research should persuade patients to re-assessment 1RM at 2-week intervals as much as possible to adjust BFRT load, thereby ensuring a standardized BFRT regimen. Furthermore, long-term follow-up and larger sample studies may be necessary to obtain more reliable findings.

## Conclusion

This study preliminarily showed that BFRT can be considered as an early routine rehabilitation program for patients after APM, which can effectively stimulate quadriceps strength and muscle hypertrophy, improve lower extremity balance, and minimize pain, thereby improving knee function. BFRT combined with routine rehabilitation training provides a promising rehabilitation training program for patients after APM.

## Data Availability

The raw data supporting the conclusions of this article will be made available by the authors, without undue reservation.
